# Assessment of resting myocardial blood flow in regions of known transmural scar to confirm accuracy and precision of 3D cardiac positron emission tomography

**DOI:** 10.1186/s13550-023-01037-7

**Published:** 2023-09-27

**Authors:** Robert M. Bober, Richard V. Milani, Sergey M. Kachur, Daniel P. Morin

**Affiliations:** 1 Department of Cardiovascular Diseases, John Ochsner Heart and Vascular Institute, Ochsner Health, 1514 Jefferson Highway, New Orleans, LA 70121-2483 USA; 2 Ochsner Clinical School, Queensland University School of Medicine, New Orleans, LA USA

**Keywords:** Positron emission tomography (PET), Myocardial scar, Resting myocardial blood flow

## Abstract

**Background:**

Composite invasive and non-invasive data consistently demonstrate that resting myocardial blood flow (rMBF) in regions of known transmural myocardial scar (TMS) converge on a value of ~ 0.30 mL/min/g or lower. This value has been confirmed using the 3 most common myocardial perfusion agents (^13^N, ^15^O-H_2_O and ^82^Rb) incorporating various kinetic models on older 2D positron emission tomography (PET) systems. Thus, rMBF in regions of TMS can serve as a reference “truth” to evaluate low-end accuracy of various PET systems and software packages (SWPs). Using ^82^Rb on a contemporary 3D-PET-CT system, we sought to determine whether currently available SWP can accurately and precisely measure rMBF in regions of known TMS.

**Results:**

Median rMBF (in mL/min/g) and COV in regions of TMS were 0.71 [IQR 0.52–1.02] and 0.16 with 4DM; 0.41 [0.34–0.54] and 0.10 with 4DM-FVD; 0.66 [0.51–0.85] and 0.11 with Cedars; 0.51 [0.43–0.61] and 0.08 with Emory-Votaw; 0.37 [0.30–0.42], 0.07 with Emory-Ottawa, and 0.26 [0.23–0.32], COV 0.07 with HeartSee.

**Conclusions:**

SWPs varied widely in low end accuracy based on measurement of rMBF in regions of known TMS. 3D PET using ^82^Rb and HeartSee software accurately (0.26 mL/min/g, consistent with established values) and precisely (COV = 0.07) quantified rMBF in regions of TMS. The Emory-Ottawa software yielded the next-best accuracy (0.37 mL/min/g), though rMBF was higher than established gold-standard values in ~ 5% of the resting scans. 4DM, 4DM-FDV, Cedars and Emory-Votaw SWP consistently resulted values higher than the established gold standard (0.71, 0.41, 0.66, 0.51 mL/min/g, respectively), with higher interscan variability (0.16, 0.11, 0.11, and 0.09, respectively).

*Trial registration*: clinicaltrial.gov, NCT05286593, Registered December 28, 2021, https://clinicaltrials.gov/ct2/show/NCT05286593.

**Supplementary Information:**

The online version contains supplementary material available at 10.1186/s13550-023-01037-7.

## Background

**Fig. 1:**
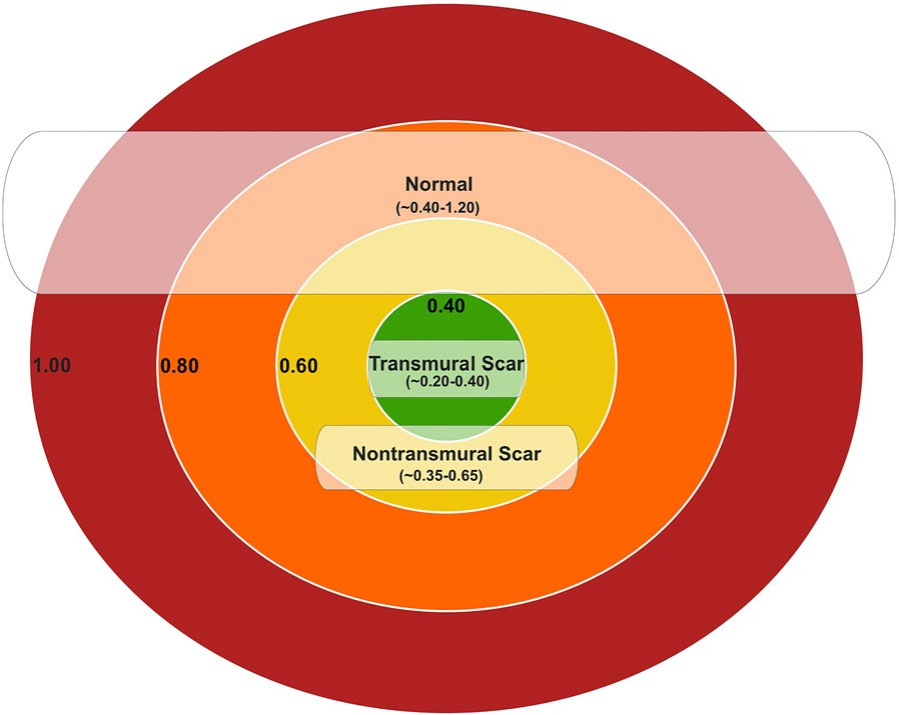
Range of resting myocardial blood flow in regions of transmural scar. The range of resting myocardial blood flow in regions of transmural scar is narrow and constrained between ~ 0.20 and 0.39 mL/min/g with a mean of ~ 0.30 mL/min/g. rMBF in non-transmural scar and normal tissue is greater and with a wider range. Thus, transmural scar can be used as a reference “truth” to assess accuracy of quantitative software packages and/or PET systems

Composite data derived from invasive, histologic, and non-invasive techniques have consistently established that mean resting myocardial blood flow (rMBF) in regions of transmural myocardial scar (TMS) converge on a value of < 0.30 cc/min/g, with mean minimum rMBF ≤ 0.20 mL/min/g and an upper bound of 0.39 [1–12]. These values have been replicated with three PET perfusion agents (Nitroge-13N ammonia [^13^N], ^15^O-H_2_O, and Rubidium-82 Chloride [^82^Rb]), as well as with microspheres [1–12]. Thus, rMBF in regions of TMS can serve as a reference “truth,” to evaluate the low-range accuracy of PET systems, software packages (SWP) and radiotracer administration, as represented in Fig. 1.

Most prior studies evaluating PET-based measurement of rMBF in regions of scar were performed on older 2D scanners using ^13^N or ^15^O-H_2_O. All newly manufactured PET cameras operate in 3D mode exclusively. While 3D PET offers several advantages, some aspects can degrade quantitative data such as scanner saturation, scatter, and high random counts with ^82^Rb, especially during the first two minutes of arterial input scanning. Furthermore, there now is a variety of analytical SWPs that may be used to assess MBF, and the performance of these packages may vary [13].

Therefore, we sought to determine whether currently available SWP operating on a modern 3D PET-CT can accurately and precisely measure rMBF in regions of known TMS (where lack of viability has already been determined). In addition, we sought to determine test–retest covariance (COV) of regional and whole heart rMBF in the same subject.

By design, we set out to measure rMBF within known scarred/nonviable/dead myocardium. We did not attempt to determine whether tissue is viable, nor to determine low-flow thresholds for viability. Rather, we were interested in assessing low-range performance of various SWPs in regions of visually obvious transmural infarct (i.e., absence of radiotracer uptake).

## Methods

The study was approved by the Ochsner Health Institutional Review Board and was registered at ClinicalTrials.gov (NCT05286593). Electronic health records were queried to identify patients with a history of a myocardial infarction and a previous cardiac PET scan demonstrating a large TMS (≥ 20% of the LV) without evidence of viability. Viability was determined either by previous PET-FDG, cardiac magnetic resonance imaging, or on clinical grounds (e.g., akinesis on echocardiography and Q-waves on electrocardiography; multiple prior non-invasive perfusion scans demonstrating a large, severe, fixed defect; or a history of ST-elevation myocardial infarction with late or no reperfusion). We identified 62 patients meeting these criteria, of whom 12 men and 10 women were selected primarily on willingness to participate and secondarily based on location of the known scar (to ensure representation of scars in various myocardial regions). All patients gave written informed consent to undergo additional PET-CT scanning for this study. PET scanning was performed between January and August 2022.

### PET protocol and image acquisition

**Fig. 2:**
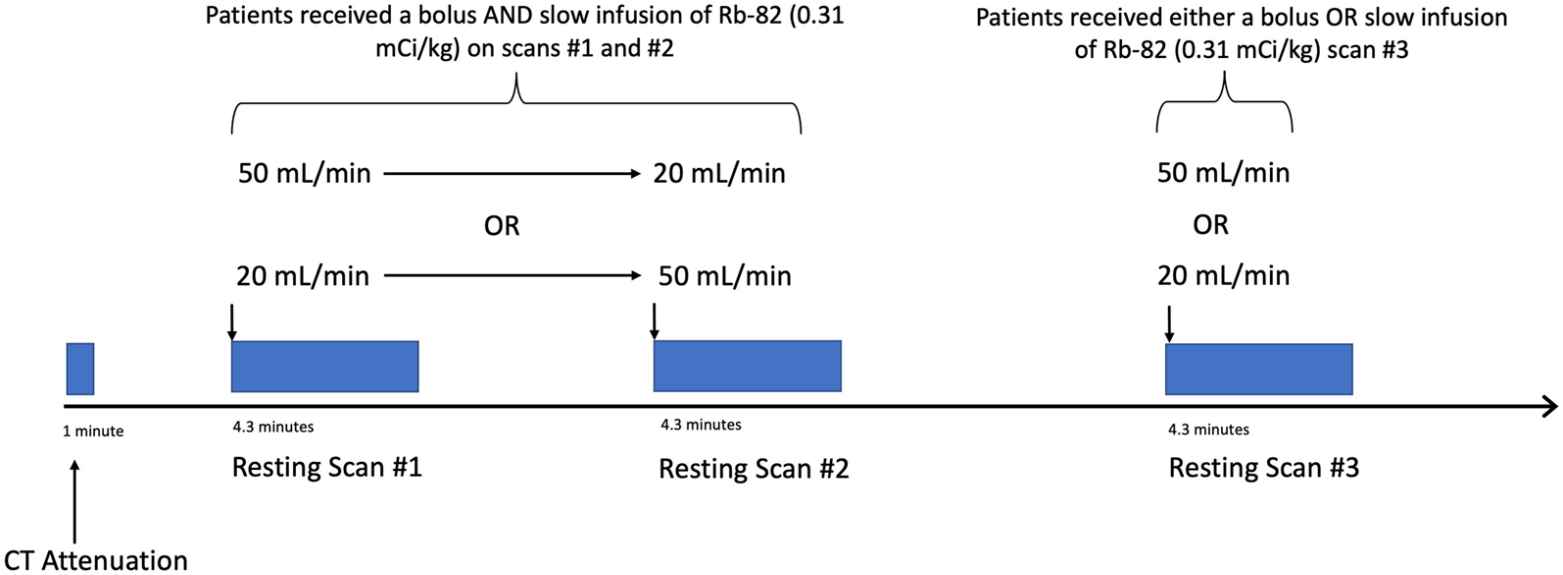
PET imaging protocol. Subjects received 3 consecutive resting ^82^Rb scans using 2 distinct infusion profiles

Prior to image acquisition, participants fasted for at least 4 h and abstained from caffeine for at least 48 h. Cardiac PET-CT was performed on a GE Discovery MI-DR PET-CT 3D scanner (GE Healthcare, Waukesha, Wisconsin) in three-dimensional mode with list mode acquisition. Each patient underwent three consecutive resting emission scans, minutes apart, each utilizing one of two ^82^Rb infusion profiles as detailed in Fig. 2. For the first two resting scans, patients sequentially received ^82^Rb as both a 50 mL/min bolus (B) and as a 20 mL/min slow infusion (SI), in random order. For the third scan, patients randomly received either a B or SI.

Emission images were obtained over 4.3 min after intravenous injection of 11.4 MBq/kg [14] of generator-produced ^82^Rb (Bracco Diagnostics, Princeton, New Jersey). The 3D list mode data were re-binned into 40 frames (28 × 5 s, 12 × 10 s), using reconstruction algorithm VuePointHD (2 iterations, 24 subsets) and a Butterworth filter (order 10, cutoff 18 mm) to generate dynamic images for MBF quantification. Random, scatter, attenuation, and decay corrections were automatically applied to the 5- and 10-s emission data. Upon arrival of activity on time activity curves, the first two minutes of the 5 s corrected emission data were summed for the retention model arterial input, and the remaining 10 s corrected frames were summed for retention model myocardial images.

### Image analysis

The 66 resting images (i.e., 3 images for each of the 22 patients) were checked for quality by several methods. First, all time-activity curves were manually inspected. Second, all images were analyzed by two different automated quality assurance (QA) algorithms [15, 16]. The QA algorithms detect possible data acquisition technical problems by identifying eight potential technical sources of error: inconsistent frame duration, scanner saturation, inaccurate blood curve peak, inappropriate blood peak width, flat blood curve tail, gradual patient motion, abrupt patient motion, and spillover fraction > 0.60.

Absolute and relative myocardial perfusion were quantified using a research version of FDA-approved software (HeartSee V3.0.0, University of Texas, Houston, TX), which employs Gould’s simplified retention model using individualized arterial inputs located in the left atrium or aorta for each resting scan, as previously described [17–19]. Partial volume loss determined with a one-dimensional tree phantom was 0.85 [20]. HeartSee (HS) has been validated for accuracy experimentally, as well as for precision in human test–retest, for correct arterial input and clinical outcomes [21]. Resting MBF and relative percent uptake (%RU) were obtained for the whole heart, for each quadrant, and for myocardial segments in a standard 17-segment model. A region of interest (ROI) was placed around the scar (“ROI-Scar”) which automatically identified the size of the scar, the average rMBF within that scar, minimum rMBF, as well as the average percent relative uptake [1]. Normally perfused myocardium not included in ROI-Scar was considered “ROI-Norm”.

Datasets also were systematically processed for analysis using three other SWP according to each vendor’s user manual: (1) 4DM V2018.0.0.226 (INVIA, Ann Arbor, Michigan), (2) Cedars-Sinai V2017.17.0.33822 (Cedars-Sinai, Los Angeles, CA) and (3) Emory Toolbox V4.2.8321.27671 (Syntermed, Anaheim, CA). 4DM and Cedars-Sinai (Cedars) employ a 1-tissue “Lortie” compartment kinetic model (1-TCM) without a fixed distribution volume. 4DM also has the option with a fixed distribution volume (4DM-FDV). 4DM and Cedars derive the arterial input from time activity curves from an ROI at the mitral valve plane. Emory utilizes the Votaw retention model (ECT-V) where the arterial input is calculated from time activity curves from a ROI within the LV cavity. We also tested a “beta” version of Emory employing the “Ottawa model” (ECT-O), which uses a 1-TCM and derives its arterial input from time activity curves via an ROI at the mitral valve plane. 4DM and ECT-O feature automatic motion correction, whereas motion correction was performed manually for Cedars. Manual adjustments to the contours and motion correction of the dynamic sequences were performed as needed. All models have been described in detailed prior publications [13, 22].

**Fig. 3:**
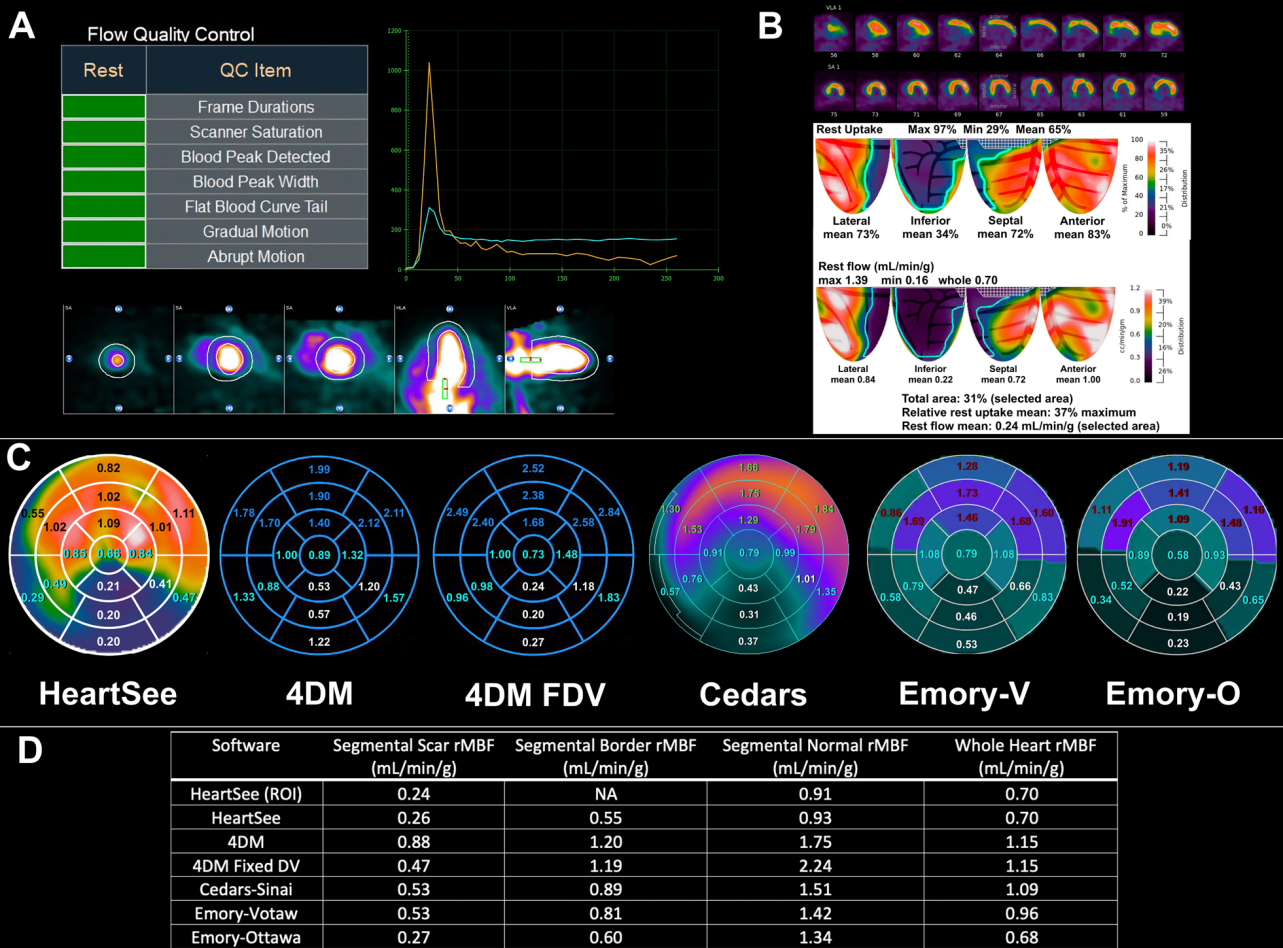
An example of the methodology in a large severe inferior transmural scar. **A** Quality assurance was checked using visual assessment of time activity curves and two automated algorithms. Motion correction was performed as needed. **B** A ROI was placed around the severe defect on relative images with the resultant size, RU% and average rMBF displayed. This ROI was defined as “ROI-Scar”. **C** Segmental analysis of the 17-segment polar maps from all SWP. Scar was defined as contiguous segments within the perfusion defect that comprised > 50% of the segment with a segmental summed rest score ≥ 2 and %RU < 65% in all four SWP. These segments are in white font and their average was defined as “Seg-Scar”. Contiguous segments involving the scar but not meeting the noted requirements were averaged and defined as “Seg-Border” (turquoise font). The remaining normal segments (various colors) were averaged and defined as “Seg-Norm”. **D** Comparisons of rMBF between ROI-Scar and Seg-Scar in various SWP

The 4DM, Cedars and Emory SWPs do not offer an ROI tool that solely selects the scar. Instead, these SWPs utilize the standard 17-segment model, in which scars may involve more than one segment and may not completely encompass any entire segment. Therefore, we identified the most severely affected segments in each scan for which all 4 SWPs demonstrated a severe perfusion abnormality, as defined by 4 criteria: (1) segments were contiguous, (2) defect encompassed > 50% of the segment, (3) segmental summed resting score was  ≥ 2, and (4) segmental %RU was < 65% maximum. Using these criteria, the segments selected for analysis were clearly severe defects in all SWP. For each scan and for each SWP, the rMBF in these segments were averaged to obtain segmental rMBF of the scar (Seg-Scar). Segments not included within Seg-Scar, but still containing a portion of the contiguous perfusion defect, were averaged to determine rMBF in the “border zone” of the infarct (Seg-Border). All other segments, which were without perfusion abnormalities, were averaged to determine “normal” rMBF (Seg-Norm). Figure 3 demonstrates an example of ROI-Scar, Seg-Scar, Seg-Border, and Seg-Norm. Thus, ROI-Scar allows for quantification confined solely to the TMS, whereas Seg-Scar allows for direct comparison between SWP.

### Statistical analysis

Statistical analysis was performed using SPSS Version 20.0 (IBM SPSS, Armonk, NY) and R Version 4.2.1 (The R Foundation for Statistical Computing, R Core Team 2021). The normality of variables’ distributions was evaluated using Q–Q plots and the Shapiro–Wilk test. Continuous and discrete data are presented as mean ± standard deviation (SD) for normal distributions, and/or median and interquartile range (IQR) when data were not normally distributed. Categorical variables are presented as frequencies and percentages. Friedman and Wilcoxon signed rank tests, as appropriate, were used to compare distributions. The coefficient of variation (COV) between scans was calculated as SD of differences divided by the mean. For all tests, two-sided *p* < 0.05 was considered statistically significant.

## Results

Technical problems were identified in images from two patients and were excluded from the final analysis. No technical problems were identified in any image from the remaining 20 patients. Sixty resting scans were analyzed from these 20 subjects. The participants’ relevant clinical history is displayed in Table 1. The mean age was 62 ± 11 years, 50% were female, mean LVEF was 37 ± 8%, mean was BMI 31.6 ± 7 kg/m^2^, and mean duration of scar was 8.6 ± 5.9 years. The locations of TMS were evenly distributed among coronary territories.

**Table 1:** Characteristics of the population

Patient	Age	Gender	LVEF	BMI	HTN	DM	HPL	CABG	Territory of scar	Duration of scar (years)
1	82	M	50	30		X	X		LCX	8
2	44	F	33	36	X	X	X		LAD	8
3	59	F	45	37	X		X		LCX	4
4	65	F	46	33	X		X		LAD	4
5	63	M	40	25	X	X	X		LAD	3
6	52	M	26	29	X	X	X		RCA	19
7	50	M	30	27		X	X		LAD & LCX	9
8	63	F	37	30		X	X		LCX	10
9	57	M	39	25			X	X	RCA	16
10	73	M	34	28	X	X	X	X	LCX	20
11	74	F	47	26	X		X	X	RCA & LCX	6
12	51	F	44	38	X		X		LAD	5
13	68	M	26	33	X		X		LAD	7
14	54	M	35	32	X				RCA	2
15	68	M	29	23	X	X	X	X	RCA	18
16	65	F	28	25	X	X	X		LAD	6
17	68	M	43	31	X	X	X		LAD	1
18	67	F	46	55	X	X		X	RCA	3
19	40	F	39	35	X	X	X		RCA	8
20	67	F	25	34	X	X		X	RCA	15

BMI = Body mass index; HTN = hypertension; DM = diabetes mellitus; HPL = hyperlipidemia

CABG = coronary artery bypass grafting

### ROI-scar results

Specific analysis of the ROI of transmural scar was performed in HS. As demonstrated in Table 2, the infarcts were large and severe (median scar size was 26% of LV myocardium, and median %RU was 43% of maximum). The median rMBF (in mL/min/g) in the ROI of transmural scar was 0.26 [0.23–0.32] and median minimum was 0.17 [0.16–0.20]. Median and mean rMBF in normal regions outside of the infarct zone were 0.69 [0.56–0.97] and 0.78 ± 0.27, respectively, and median whole-heart rMBF was 0.62 [0.47–0.83]. The COV was 6–8% for all rMBF measurements.

**Table 2:** Analysis of region of interests of transmural scar (ROI-Scar) in HeartSee

	ROI-Scar size (%LV)	ROI-Scar %RU	ROI-Scar rMBF (mL/min/g)	Minimum rMBF (mL/min/g)	ROI-Norm rMBF (mL/min/g)	Whole heart rMBF (mL/min/g)	ROI-Scar COV	ROI-Norm COV	Whole heart COV
Mean ± SD	27 ± 9	43 ± 4	0.27 ± 0.05	0.18 ± 0.05	0.78 ± 0.27	0.65 ± 0.21	0.07 ± 0.04	0.08 ± 0.04	0.08 ± 0.05
Median [IQR]	26 [20–31]	43 [40–45]	0.26 [0.23–0.32]	0.17 [0.16–0.20]	0.69 [0.56–0.97]	0.62 [0.47–0.83]	0.06 [0.05–0.08]	0.07 [0.06–0.12]	0.06 [0.05–0.12]

**Fig. 4:**
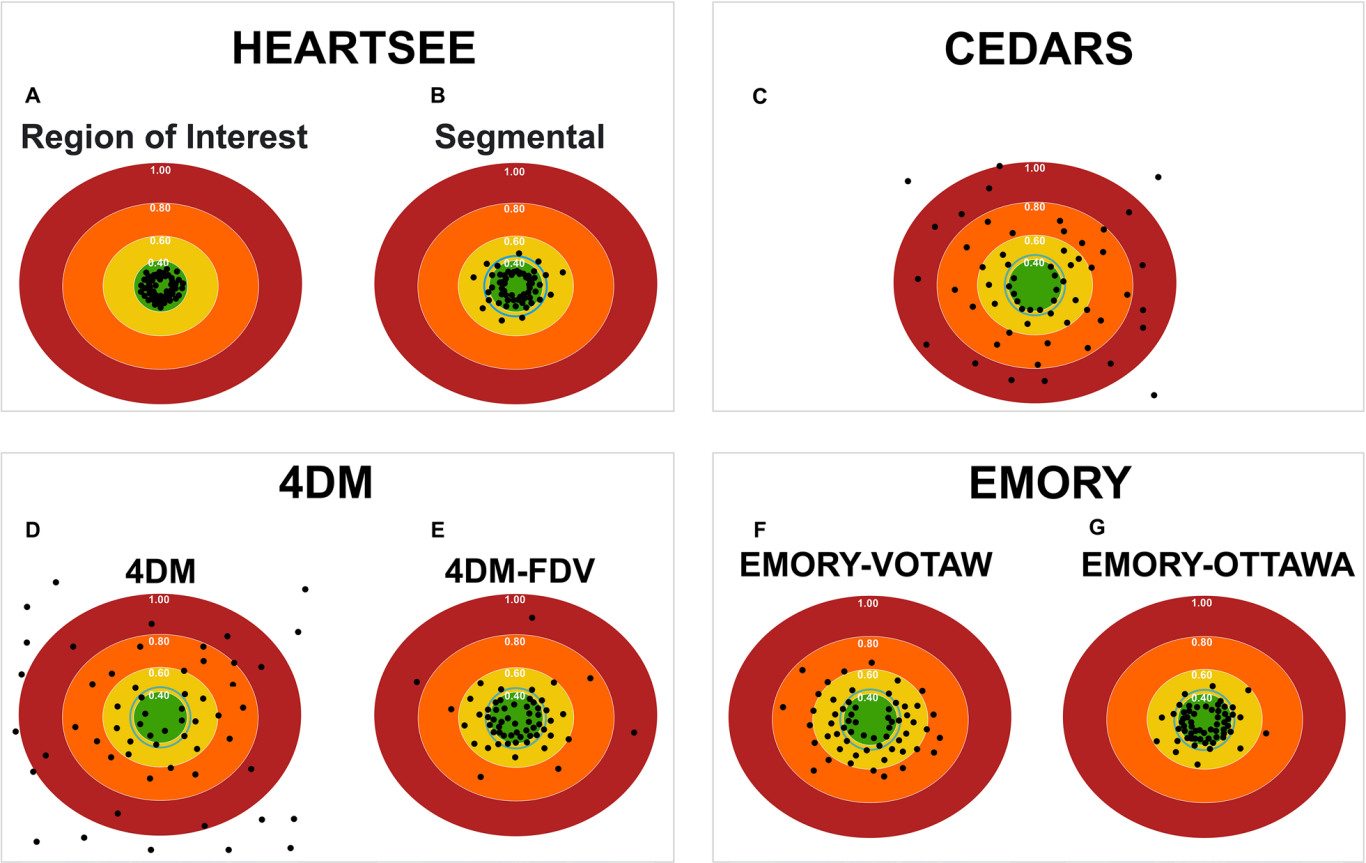
Accuracy of resting MBF of transmural scar per software package. The green center represents the expected rMBF of transmural scar (< 0.39 mL/min/g for ROI analyses). The layered concentric circles outside the green center represent higher rMBF. The blue concentric circle immediately outside the green circle is for segmental analyses limited by the upper bounds of 0.44 mL/min/g. For each software package, the computed rMBF for infarcts from all 60 resting scans are displayed as round dots. All values are in mL/min/g

As a metric of accuracy and upward bias, the upper limit threshold of rMBF in TMS was 0.39 mL/min/g, which is 1SD above the mean of rMBF within TMS as has been established in the literature for a ROI of TMS (Fig. 4a) [1]. All 60 ROI-Scars appropriately had mean rMBF less than 0.39 mL/min/g. There was no significant difference in any rMBF metric between scans using a ^82^Rb bolus at 50 mL/min infusion versus a slow infusion at 20 mL/min. (ROI-Scar rMBF 0.25 [0.22–0.30] vs. 0.28 [0.24–0.32], *p* = 0.161; minimum rMBF 0.16 [0.14–0.19] vs. 0.17 [0.16–0.20], *p* = 0.136; ROI-norm rMBF 0.66 [0.53–0.85] vs. 0.79 [0.58–1.00], *p* = 0.166; and whole heart rMBF 0.57 [0.44–0.70] vs. 0.70 [0.49–0.84], *p* = 0.125).

### Region of interest versus 17-segment model (ROI-Scar vs. Seg-Scar) in HeartSee

**Fig. 5:**
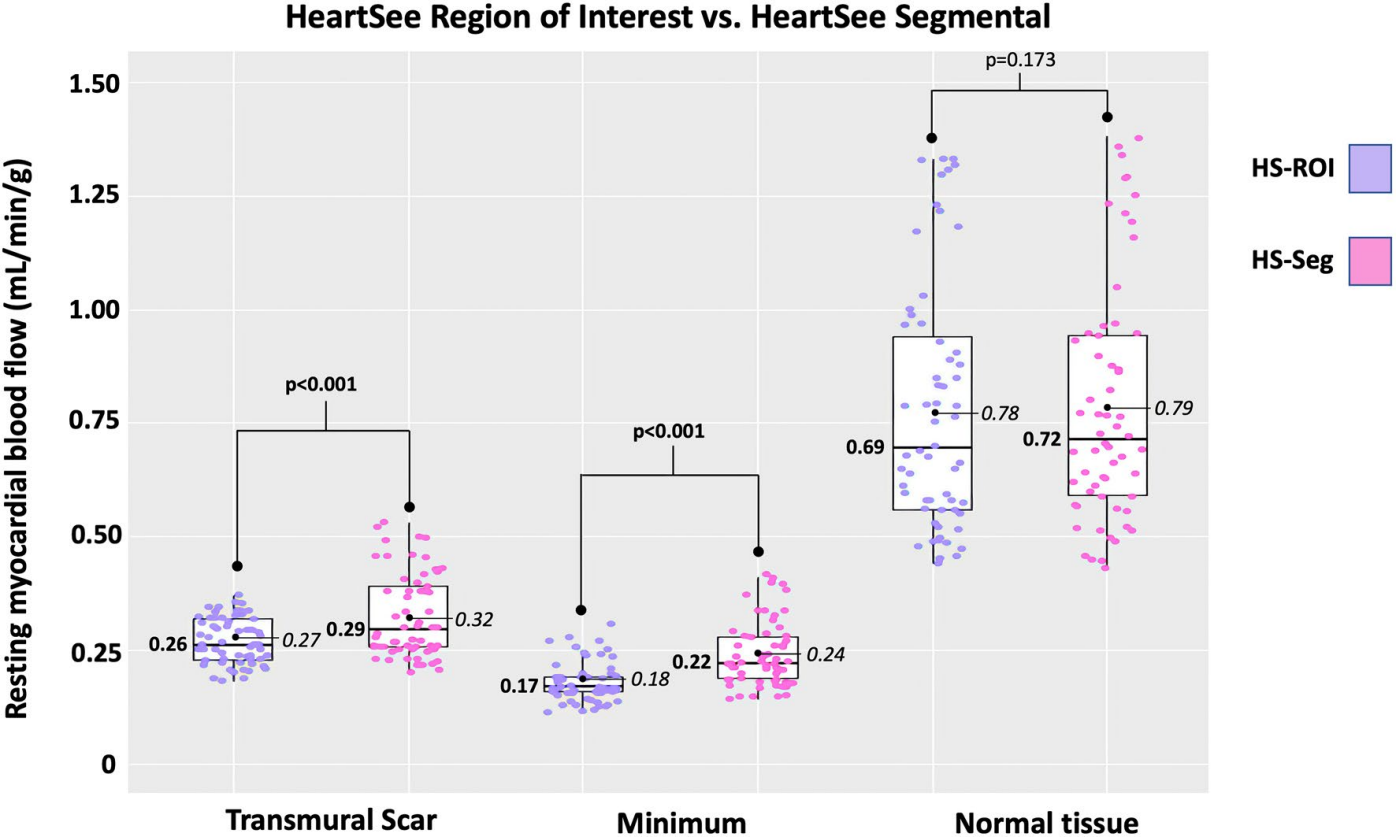
Comparison of HeartSee ROI-Scar versus Seg scar

As demonstrated in Fig. 5 and Table 3, median rMBF was significantly lower in ROI-Scar: 0.26 [0.23–0.32] versus Seg-Scar 0.29 [0.26–0.40] mL/min/g, *p* = 0.001. %RU was lower in ROI-scar versus Seg-Scar: 43% [40–45] versus 46% [42–49], *p* < 0.001. As a metric of accuracy and upward bias, the upper limit threshold of rMBF in Seg-Scar was 0.44 mL/min/g which is the maximum 1SD above the mean of rMBF within TMS as has been established in the literature for a segment of TMS [2, 6, 8, 12]. When ROI-Scar was analyzed, all 60 scans returned rMBF lower than 0.39 mL/min/g. When Seg-Scar was analyzed, 9 of 60 (15%) scans returned a scar-related rMBF > 0.44 mL/min/g (Fig. 4b). There were 235 myocardial segments associated with scar among the 60 resting scans. Among these, 36 (15%) segments had rMBF above the established acceptable upper limit of 0.44 mL/min/g.

**Table 3:** Comparison of HeartSee ROI-Scar versus Seg scar

	Scar		*p*-value	Minimum		*p*-value	Normal		*p*-value
	ROI	Seg		ROI	Seg		ROI	Seg	
Mean ± SD	0.27 ± 0.05	0.32 ± 0.09		0.18 ± 0.05	0.24 ± 0.07		0.78 ± 0.27	0.79 ± 0.26	
Median [IQR]	0.26 [0.23–0.32]	0.29 [0.26–0.40]	< 0.001	0.17 [0.16–0.20]	0.22 [0.19–0.28]	< 0.001	0.69 [0.56–0.97]	0.72 [0.59–0.99]	0.173

### Comparison of segmental analysis among software packages

**Fig. 6:**
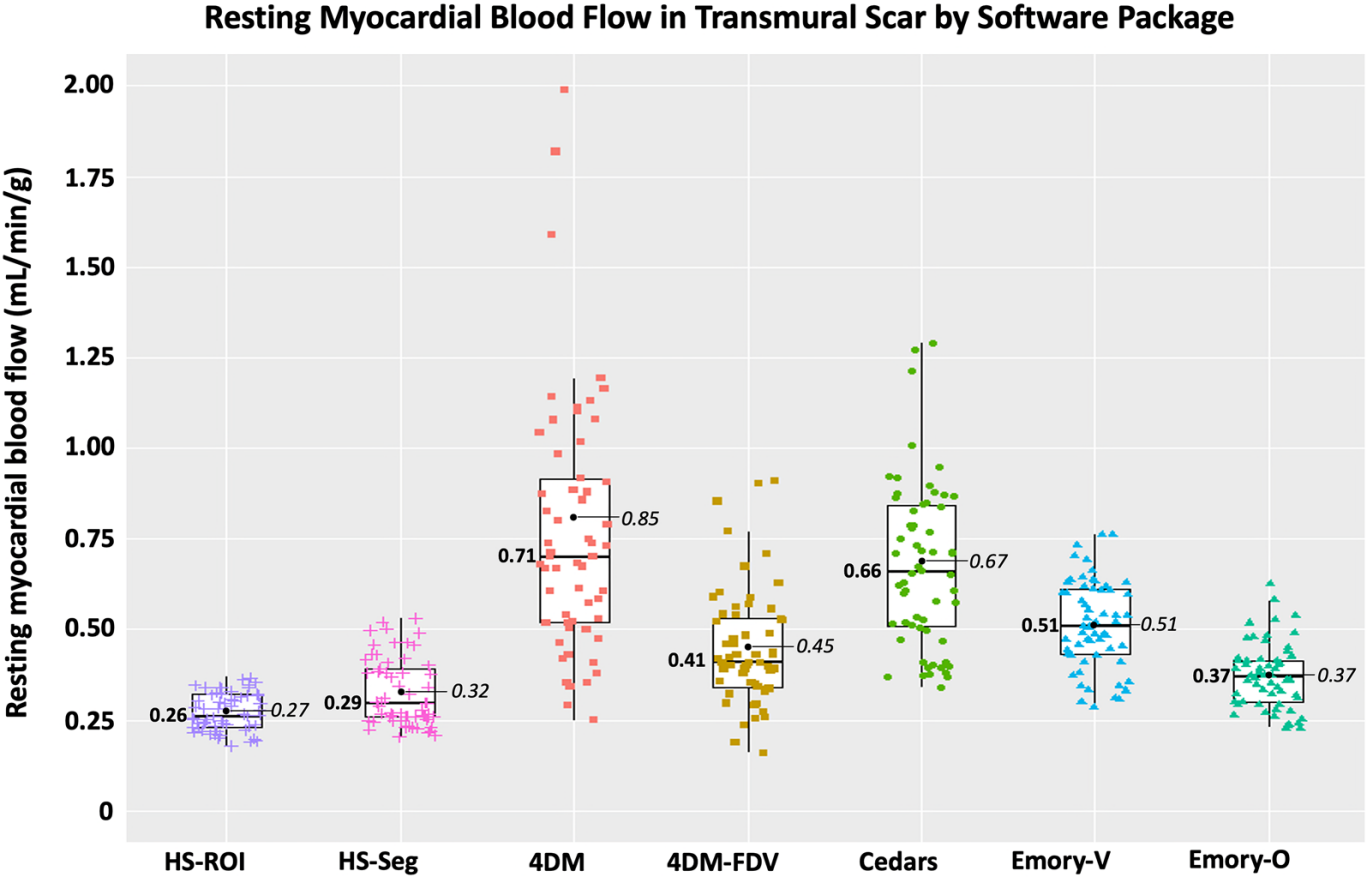
Resting MBF in transmural scar by software package. All analyses are segmental apart from HS-ROI. HS = HeartSee, 4DM-FDV = 4DM with fixed distribution volume, Emory-V = Emory Votaw model, Emory-O = Emory Ottawa model

**Fig. 7:**
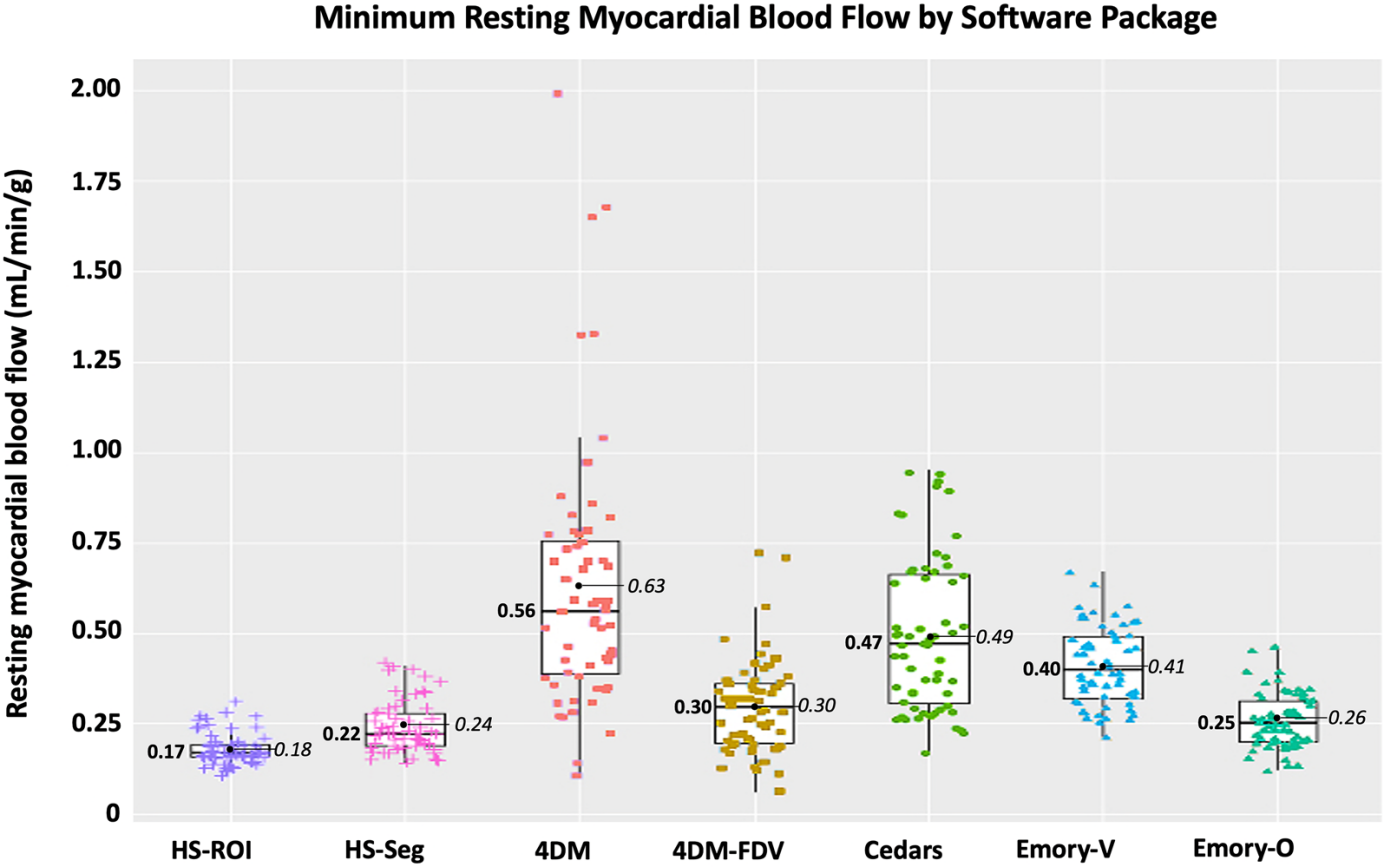
Minimum resting MBF within transmural scar by software package

**Fig. 8:**
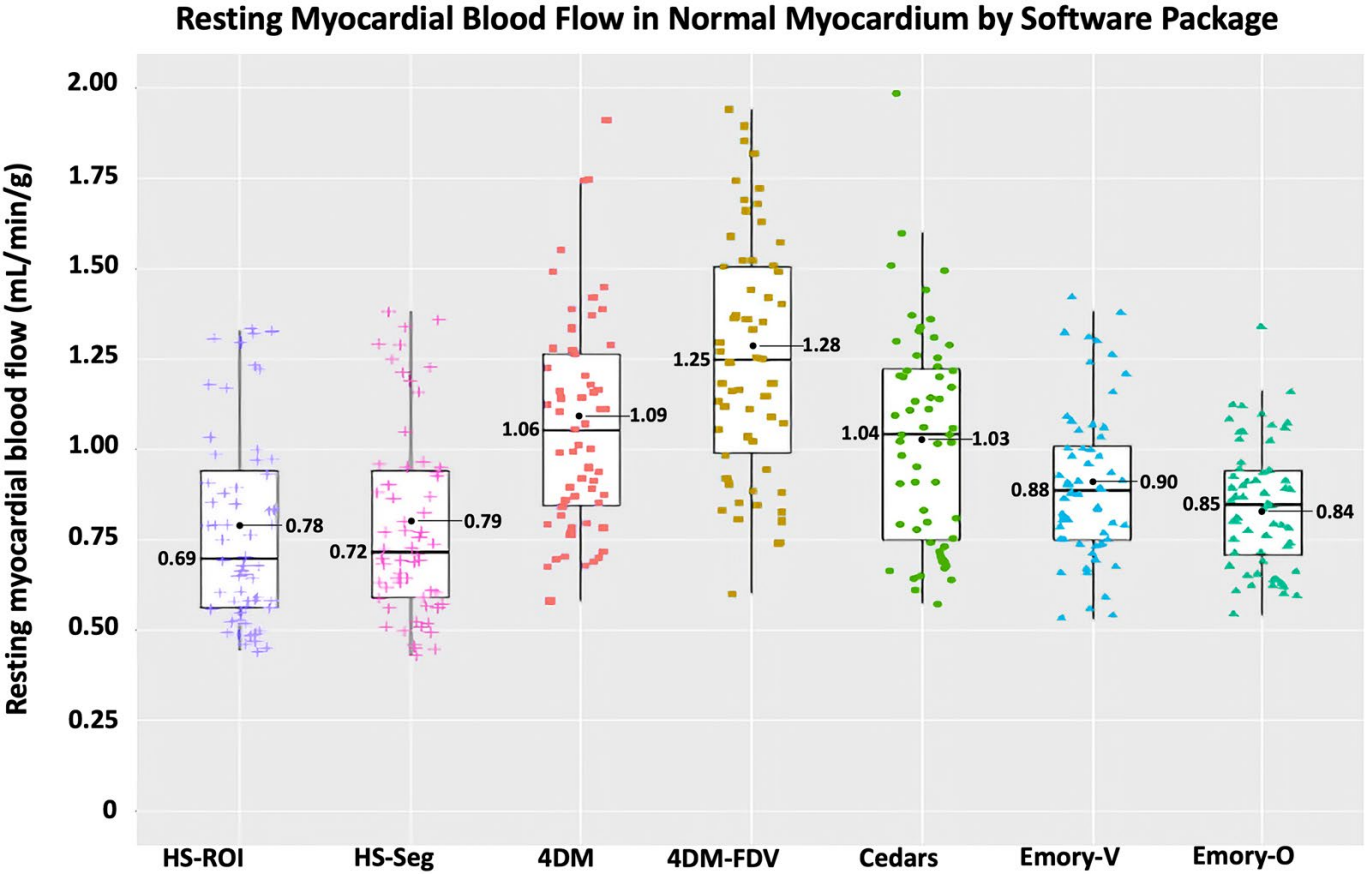
Segmental resting MBF of normal myocardium surrounding transmural scar by software package

**Fig. 9:**
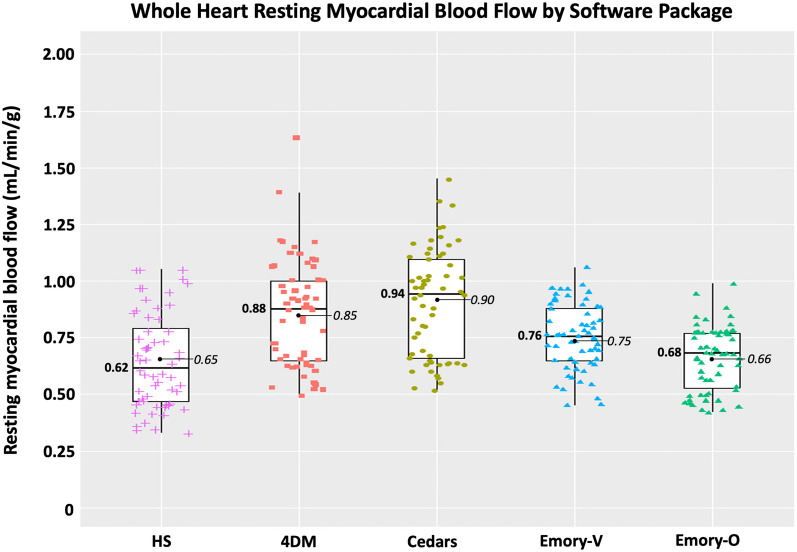
Whole heart resting MBF of patients with transmural scar by software package

In the 60 resting scans analyzed, a total of 235 segments were associated with scar, which involved an average of 3.9 ± 1.4 segments per scan. As depicted in Figs. 4 and 6 and Table 4, there are several important associated findings. First, compared with Seg-Scar measured with HeartSee, all other SWP reported significantly higher rMBF in Seg-Scar. Second, all SWP except HS and ECT-O commonly reported rMBF values inconsistent with transmural infarct. As demonstrated in Figs. 7, 8 and 9 and Table 4, the same pattern was found (i.e., all SWP had higher rMBF than HS) for Seg-Minimum, Seg-Norm, and Whole Heart rMBF. The only exceptions to this pattern were with HS-Seg-Minimum versus Emory-O for Seg-Minimum and whole heart rMBF, for which there was no significant difference (Additional file 1: Tables S1 and S2).

**Table 4:** Segmental analysis of rMBF of and surrounding transmural scar by software package

SWP	rMBF in Seg-Scar (mL/min/g)	Lowest segment (mL/min/g)	rMBF in Seg-Border (mL/min/g)	rMBF in Seg-Normal (mL/min/g)	Whole heart rMBF (mL/min/g)	Size of Scar (% LV myocardium)	COV Scar	%Relative uptake
HeartSee	0.29 [0.26–0.40]	0.22 [0.19–0.28]	0.48 [0.43–0.65]	0.65 [.52–0.91]	0.62 [0.47–0.83]	26 [20–31]	0.07	0.46‡ [0.42–0.49]
4DM	0.71 [0.52–1.02]	0.56 [0.39–0.77]	0.82^*^ [0.70–1.05]	1.02 [0.78–1.20]	0.88^†^ [0.65–1.00]	26 [20–35]	0.16	0.47‡ [0.44–0.49]
4DM-FDV	0.41 [0.34–0.54]	0.30 [0.20–0.37]	0.82^*^ [0.67–1.00]	1.17 [0.91–1.41]	0.88^†^ [0.65–1.00]	26 [20–35]	0.11	0.47‡ [0.44–0.49]
Cedars	0.66 [0.51–0.85]	0.47 [0.31–0.67]	0.87^*^ [0.69–1.05]	1.00 [0.72–1.24]	0.94^†^ [0.66–1.11]	25 [19–30]	0.11	0.41 [0.40–0.44]
Emory-V	0.51 [0.43–0.61]	0.40 [0.32–0.50]	0.70 [0.60–0.84]	0.84 [0.73–0.98]	0.76 [0.65–0.88]	26 [21–32]	0.09	0.45‡ [0.41–0.48]
Emory-O	0.37 [0.30–0.42]	0.25 [0.20–0.32]	0.64 [0.49–0.73]	0.80 [0.66–0.90]	0.68 [0.53–0.77]	26 [21–32]	0.08	0.45‡ [0.41–0.48]
*p*-value	< 0.001	< 0.001	< 0.001	< 0.001	< 0.001	0.779	< 0.001	< 0.001

*4DM versus 4DM-FDV versus Cedars—*p* = 0.118

^†^4DM versus 4DM-FDV versus Cedars—*p* = 0.105

^‡^HS versus 4DM versus 4DM-FDV versus Emory-V versus Emory-O—*p* = 0.077

Of the 60 scans, the number of scans with Seg-Scar with mean rMBF above 0.44 was 9 (15%) with HS; 51 (85%) with 4DM, 23 (38%) with 4DM-FDV, 48 (80%) with Cedars, 42 (70%) with ECT-V, and 12 (20%) with ECT-O (Fig. 4). Out of the 235 segments, the number of segments greater than 0.44 cc/min/g was 36 (15%) with HS, 177 (75%) with 4DM, 87 (37%) with 4DM-FDV, 168 (71%) with Cedars, 150 (64%) with ECT-V and 54 (23%) with ECT-O.

Finally, there was no statistical difference in any rMBF metric between a bolus infusion at 50 mL/min ^82^Rb versus a slow infusion at 20 mL/min for each SWP (Additional file 1: Table S3).

## Discussion

This study has several important findings. First, we demonstrate that SWPs vary widely in low end accuracy based on measurement of rMBF in regions of known TMS. Second, we confirm the “low flow” accuracy and test–retest precision of a modern 3D PET-CT, via examination of a reference region of dense TMS using the software package HeartSee. Third, we describe a method of “converting” a ROI of TMS to segmented ROI and provide reference values whereby myocardial blood flow within transmural infarctions can be easily measured even within the significant limitations of the standard 17-segment model. Fourth, we report that while HS and, for a large extent, Emory-O, yielded values consistent with transmural scar, other SWPs consistently overestimated rMBF within dense scar. Our data also provide insights into the mechanism(s) for these SWPs’ upward biases. Finally, we establish that the same-day test–retest variability of rMBF in patients with TMS is ~ 7–9% using a modern 3D PET-CT.

### Transmural scar as a reference of “truth”

There are numerous publications comparing various quantitative SWP. However, when between-SWP discordances are present, the user is left uncertain which (if any) is accurate [13, 23–26]. Current recommendations that suggest each laboratory set their own thresholds for quantification [27, 28] are antithetical to the very nature of quantitative PET [29]. The question remains, “What simple means exist for PET laboratories to determine accuracy of SWP?”.

When evaluating the performance of quantitative PET imaging, a variety of test cases are available to clinicians, with various flow characteristics: (1) “normal” MBF in healthy volunteers (2) “ischemic” sMBF and (3) rMBF in transmural scar. “Normal” MBF flow is too heterogenous and with standard deviations too wide to be used as a reference, whereas “ischemic” flow values also will vary depending upon the definition of “ischemia” [30, 31]. However, resting flow in transmural scar is not physiologically burdened with this wide variability, and has several other favorable characteristics. First, by definition, the myocardium within the transmural scar is dead, which can be confirmed with adjunct advanced testing such as MRI or FDG or based on history and common clinical studies (i.e., echocardiography, ECG, SPECT). Second, TMS is easy to identify, as there is a severe defect within the region of analysis. Gupta et al., for example, have used a threshold of ≤ 50% maximum uptake as a definition of transmural scar [32]. As noted, Table 2 demonstrates that the mean uptake of infarcts in the current study was 41–47% for all SWPs. Third, rMBF within the scar (by definition) should be lower than living viable tissue. Various studies authored by Beanlands et al., Benz et al., Wang et al., and Zhang et al. determined viability rMBF “thresholds” of 0.45, 0.45, 0.42, and 0.42 mL/min/g, respectively [2, 10, 12, 33]. Below these thresholds, non-viable, dead myocardial scar must be present.

However, these noted studies did not discriminate scar thickness (i.e., between transmural and non-transmural scar). In one of the most compelling studies on this topic, Rivas et al. [3] performed a transmural analysis with microspheres from endocardial through epicardial layers in infarcted dogs. They found a gradient of rMBF ranging from 0.00 to 0.35 mL/min/g in layers of myocardium with scar thickness > 72%. Furthermore, as scar thickness decreased (non-transmural scar became predominant), rMBF increased (rMBF ranged from 0.36 to 0.75 mL/min/g in non-TMS). The greater the scar thickness, the lower the rMBF [3]. Similar to Rivas, Stewart et al. also demonstrated an inverse relationship between scar thickness and rMBF. They found that mean rMBF within non-TMS was 0.45 ± 0.14 mL/min/g and within TMS was 0.32 ± 0.07 mL/min/g. In this study, cardiac MRI was used to determine scar thickness and PET was used to determine rMBF in a ROI of scar. Thus, as myocardial scar thickness increases, not only does the mean rMBF decrease but also does its standard deviation. Grönman et al. [34] made similar observations with [O-15]H_2_O. However they did not specifically distinguish between TMS and non-TMS, but did find a mean rMBF of 0.45 mL/min/g. Examples within their report demonstrate rMBF < 0.30 mL/min/g within the most severe segments of scar [34].

Based on the constellation of data, reference values for rMBF within regions of TMS should not be in dispute as they were validated by invasive, histologic, and non-invasive means and summarized in Table 5. These reference values has been verified not only by PET using various radiotracers and kinetic models (^15^O-water, ^13^N ammonia, and ^82^Rb) in both human and animal studies, but also using the gold standard: microspheres [1–12]. The results of these studies utilizing multiple techniques, models, and radiotracers all consistently converge on a narrow range of 0.30 mL/min/g or less, with an upper limit of 0.44 mL/min/g (when using a segmental analysis), thus suggesting a universal limit, reference range, or “gold standard.” As a firmly established constant, all combinations of techniques, correctly functioning scanners (2D or 3D), kinetic models, and radiotracers should describe TMS rMBF within this narrow reference range. In contrast, measurements outside of this established reference range (i.e., rMBF of 0.5 mL/min/g or higher) would suggest an upward bias of the SWP, inaccuracies of the imaging system, or perhaps imaging non-TMS.

**Table 5:** Literature review of resting MBF in transmural infarct

Author	Radiotracer or method	Mean rMBF in transmural scar (mL/min/g)	Upper limit of rMBF in scar (mL/min/g)	Number of subjects or segments	Method	Camera type	Year of publication
Rivas [3]	Microspheres	Infarcted layers with rMBF ranging 0.00–0.35	0.35	11 Dogs	Direct	NA	1976
Savage [4]	Microspheres	Infarcted layers with rMBF ranging 0.06–0.25	0.25	11 Pigs	Direct	NA	1981
de Silva [5]^§^	[O-15]H_2_O	0.28 ± 0.07	0.35	12 Patients	ROI	2D PET	1992
Czernin [6]	N-13	0.32 ± 0.12	0.44	13 Patients	Segmental	2D PET	1993
Bol [7]	microspheres, N-13 and [O-15]H_2_O	0.26–0.35	0.35	6 Dogs	Direct and ROI	2D PET	1993
Gewirtz [8]	N-13	0.27 ± 0.17	0.44	22 Infarcted zones	Segmental	2D PET	1994
Sun [9]	N-13	0.28 ± 0.09	0.37	16 Patients	ROI	2D PET	1996
Beanlands^*^ [10]	N-13	0.30 ± 0.06	0.36	8 Patients	ROI	2D PET	1997
Iida [11]	Microspheres, [O-15]H_2_O	0.19 ± 0.14	0.33	12 Dogs	Direct, ROI	2D PET	2000
Zhang [12]	N-13	0.32 ± 0.09	0.41	36 Regions	Segmental	2D PET	2013
Wang [2]	N-13	0.27 ± 0.06	0.33	115 Segments (~ 8 patients)	Segmental	3D PET	2020
Stewart [1]	Rb-82	0.32 ± 0.07	0.39	16 Patients	ROI	2D PET	2022
Bober	Rb-82	0.27 ± 0.05	0.33	20 Patients–60 scans	ROI	3D PET	2023

*Two data points excluded due to residual viability

^§^Reported resting myocardial blood flow in perfusable tissue values were converted to resting myocardial blood flow per mass in total tissue by multiplying the perfusable tissue index (PTI) to the reported values in the conclusions. This allows for direct comparison between [O-15]-H20 and ^13^N and ^82^Rb [35]

Thus, rMBF within dense TMS is an optimal reference for determining software bias, because its true value is within a known narrow margin and regions of TMS are easily identified in clinical practice.

It is worth reiterating that we selectively imaged regions of myocardium with dense transmural scar, where all questions regarding any possibility of viability had been previously settled. We were not testing any hypotheses for a possible rMBF threshold for viability, nor did we focus on any region with non-transmural scar. All scans demonstrated a near absence of radiotracer in the region of TMS, and each participant’s clinical history was consistent with a large, dense, transmural scar without evidence of viability. Thus, every patient who underwent PET/CT scanning for this study had established transmural scar without any evidence of viability.

#### Difference in performance between commercial software packages

Performance differences between SWP have been a prominent topic in the literature, but “the absence of a gold standard by which to judge…accuracy” has been a major limitation [36]. Kamphuis et al. have proposed an advanced phantom pump to establish a “ground truth validation of absolute MPI applications in the clinical setting” [37]. While Bui et al. [17] have used such a phantom pump in establishing a “ground truth” for the arterial input function, their method does not test *implementation* of other aspects of kinetic models such motion, spillover, and boundary segmentation. Furthermore, such phantom pumps are not readily available, are expensive, and require a level of expertise that is beyond the capacity of most PET labs. In this study, we employed a simple inexpensive method for assessing PET systems and SWP low-end accuracy in clinical settings- namely a dense region of transmural myocardial scar without evidence of viability. Furthermore, most cardiac PET laboratories can utilize the methods described here, without additional equipment, complex pumps, or involvement in research protocols.

Using HeartSee on a contemporary 3D PET-CT, we report that the median rMBF in a ROI of TMS is 0.26 mL/min/g, with median minimal rMBF of 0.17. These expected values suggest that both camera and software are functioning appropriately.

As noted, HS provides a ROI tool such that the exact contours of the infarct can be selected by software algorithm. However, the remaining tested SWPs did not have such a feature. Thus, the only method for detailed defect analysis in non-HS SWPs was via the standard 17-segment model. The 17-segment model is suboptimal for the required analysis as most scars affect only parts of individual segments, thus biasing readings toward higher rMBF values. Therefore, to uniformly compare all SWPs, we determined a segmental equivalent (Seg-Scar) to the ROI of TMS (ROI-Scar) and provided reference values whereby transmural infarctions can be measured within the limitations of the non-physiologic segmentation employed by the standard 17-segment model. Based on our methods, median rMBF (mL/min/g) in a “segmented” TMS is 0.29 [0.26–0.40], with the lowest segment measuring 0.22 [0.19–0.28] and with an expected ~ 15% of segments showing rMBF > 0.44 (Figs. 4b and 6). Until other SWPs implement an ROI tool, our segmental methodology may be used, as it is easy to employ and functional.

In contrast to HeartSee and Emory-O, the other SWPs demonstrated consistent upward bias of rMBF for scar-related rMBF. Among the 60 scans in this study, 4DM, 4DM-FDV, Cedars-Sinai and Emory-V returned rMBF values consistent with TMS (i.e., < 0.44 mL/min/g) in 15%, 62%, 20% and 30% of scans, respectively.) Median rMBF (mL/min/g) in Emory-O Seg-Scar was significantly higher than HeartSee Seg-Scar (0.37 vs. 0.29, *p* = 0.006); however, there was only a 5% difference between the two SWP in values inconsistent with TMS. One could hypothesize that if Emory-O had an ROI tool, ~ 5% of cases would fall outside of expected values. Perhaps with incorporation of an ROI tool, this hypothesis could be tested.

Although there have been numerous publications comparing various SWPs, scant literature evaluates accuracy or rMBF within transmural scar. Benz et al. reported the median rMBF in regions of scar was 0.68 mL/min/g [0.54–0.88] using PMOD software (Version 3.7; PMOD Technologies Ltd., Zurich, Switzerland) [33]. Although we did not specifically test PMOD, it has been shown to be highly correlated with 4DM and Cedars [13, 16]. As previously shown in Table 4, Seg-Scar in 4DM and Cedars rMBF (mL/min/g) was 0.71 [0.52–1.02] and 0.66 [0.51–0.85], respectively—findings that are nearly identical to that reported by Benz. Furthermore, also as shown in Table 4, median rMBF in normal tissue using 4DM, 4DM-FDV and Cedars was ~ 1.00–1.17 mL/min/g. Numerous other publications demonstrate rMBF in “normal” tissue using 4DM and Cedars to be 1.0–1.34 mL/min/g [13, 23, 25, 38]. The combination of these comparison findings substantiate that our methodology was accurate and not biased by user error or camera type.

**Fig. 10:**
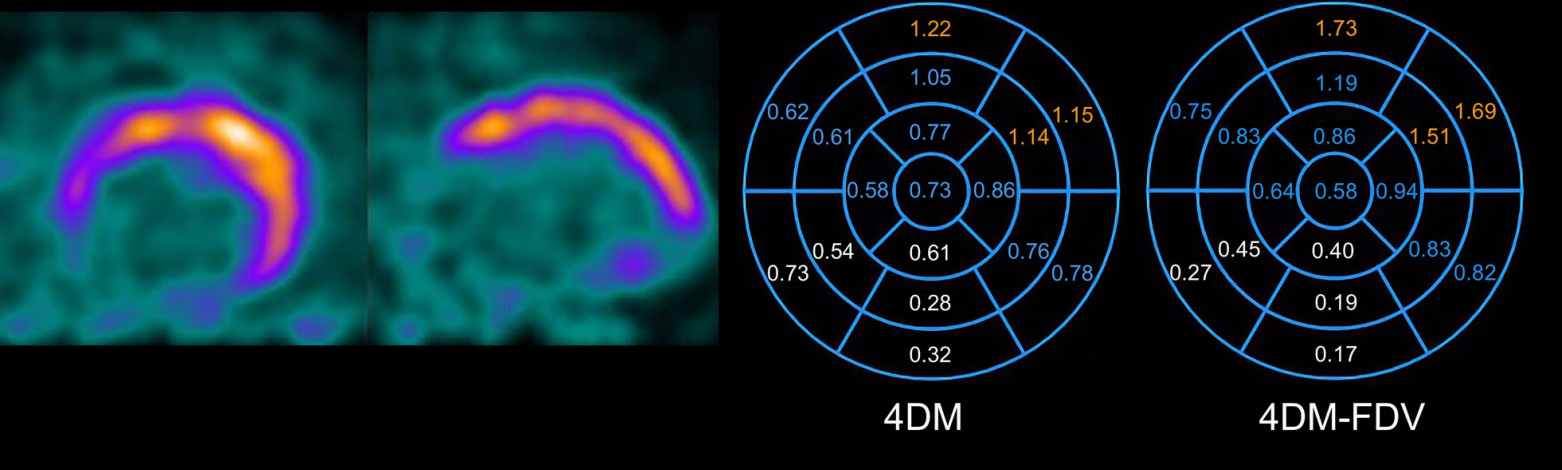
Comparison of resting MBF in 4DM versus 4DM-FDV. rMBF (mL/min/g) in the infarcted segments (white values) is 0.50 versus 0.30, respectively. This fact confirms accuracy within the infarcted segments with 4DM-FDV. However, outside of the infarcted segments, most rMBF are higher in the FDV model. The orange segments represent rMBF that are unrealistic in the 4DM-FDV model

4DM offers two options for MBF calculation using a 1-tissue compartment model (with and without an FDV). Interestingly, while Seg-Scar was significantly higher with 4DM than 4DM-FDV, whole heart rMBF was identical. To produce these findings, regions with normally perfused tissue were assigned much higher values (often unrealistically so) with FDV, as depicted in Figs. 8 and 10 and Table 4. Thus, although 4DM-FDV demonstrated less upward bias than 4DM within the region of infarct (i.e., 62% vs. 15%), rMBF outside of the infarct zone appears biased.

#### Potential sources of rMBF upward bias

As stated in the Methods section, the quality of the dynamic PET studies was deemed adequate both manually and by 2 separate automated processes. Hence, the input datasets were of good quality. As MBF = *k* * myocardial uptake/arterial input, there are 3 potential causes of upward bias: (1) invalid partial volume correction factors (one component of the variable “*k*”), (2) erroneous vendor-specific myocardial uptake processing (e.g., inaccurate motion corrections, inaccurate myocardial boundary selection, etc.), and (3) underestimated arterial input.

The methods for partial volume corrections for 4DM, Cedars and Emory-V software packages are not visible or modifiable by the user. Therefore, it is plausible that these SWP have overestimated the PV loss causing an upward bias in rMBF.

**Fig. 11:**
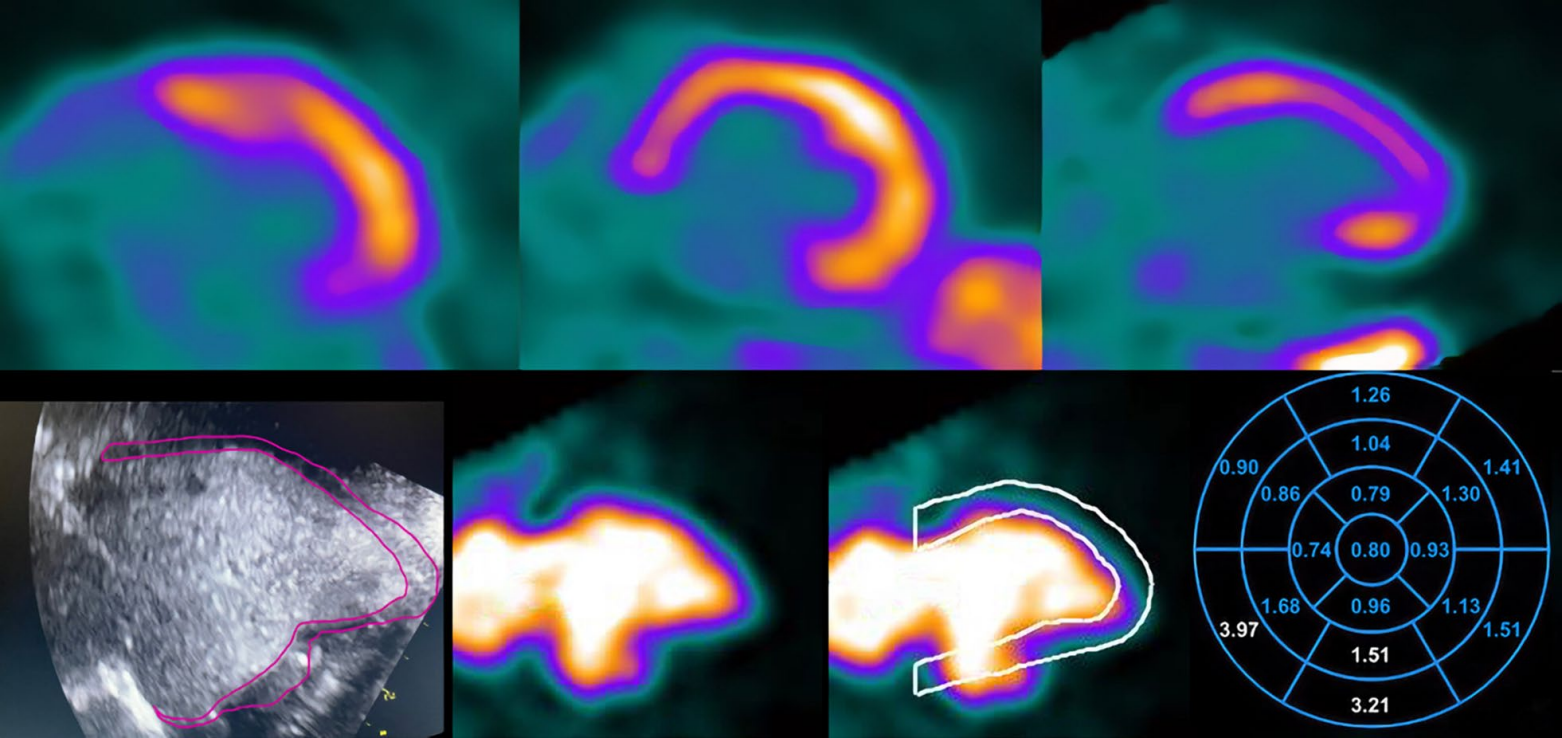
Inaccurate boundary selection. Due to the absence of myocardial uptake and LV aneurysmal segments within the transmural scar, the software package inaccurately identified myocardial boundaries, an error which could not be remedied manually with standard tools within the SWP. This erroneous boundary detection led to a “spillover effect,” yielding falsely elevated MBF values in the scar. Bottom left corner is an echocardiogram with the LV aneurysm outlined in pink

Another possibility for upward biased rMBF in TMS is software-specific erroneous myocardial uptake processing. All SWPs demonstrated nearly identical findings on relative perfusion (myocardial uptake) in terms of %RU and infarct size (all confirming visually obvious large, severe defects as demonstrated in Table 1 and Additional file 1: Table S4). However, we did find several examples of erroneous myocardial processing with 4DM and Cedars as demonstrated in Fig. 11. Due to the absence of myocardial uptake and LV aneurysmal segments within the TMS, the SWP inaccurately identified myocardial boundaries. This error could not be remedied manually with standard tools within the SWP. This erroneous boundary detection led to a “spillover effect,” yielding falsely elevated MBF values in the scar. This error did not occur with Emory-V, Emory-O or HS which implement both retention and compartmental kinetic models. Hence, we can conclude that in these situations, the upward bias was caused by vendor-specific errors in implementation of the kinetic model and not within the PET dataset. However, these examples were rare, occurring in less than 10% of the studies.

As very low MBF is proportional to the ratio of myocardial uptake to the arterial input function (tracer roll-off does not happen until higher flows), reduced %RU (as seen with the Cedars SWP) should yield lower MBF values. As this was not the case for 4DM, Cedars or Emory-V, the logical explanation for routinely upwardly biased MBF data is underestimated arterial input by the software package. In fact, this is the most logical explanation given the data we collected. Unfortunately, testing arterial input is technically challenging, not practical, and there are no standardized methods for doing so. Bui et al. [17] have demonstrated accuracy of arterial input with 2D and 3D scanners with the use of a phantom pump using a simplified retention model. However, testing of various software which utilize a compartmental model was not performed. Furthermore, it is hypothesized that a standard “one size fits all” location for the arterial input ROI (as used by 4DM, Cedars and Emory-V) is inadequate and individualized placement is necessary to achieve accuracy and consistency in perfusion metrics [17, 39].

Therefore, we can state that in a small proportion of cases in 4DM and Cedars, the upward bias of rMBF in TMS was due to inaccurate myocardial boundary detection. In the vast majority of cases in which upward bias was present, the cause could be due to erroneous PV correction, but the more likely cause is underestimated arterial input.

#### Test/retest variability

Finally, whole heart COV, and COV within scar and normal regions, was ~ 6–9% using HS, which is slightly lower but comparable to prior findings by Kitkungvan et al. with scans performed on a 2D scanner [40]. All SWP demonstrated COV ~ 10%, with the exception of 4DM (Table 4). Thus, same-day test/re-test methodologic precision has not changed significantly with the advent of contemporary 3D PET-CT systems.

#### Clinical implications

If CAD outcomes (e.g., death and spontaneous MI) are modifiable with revascularization, we postulate that specific actionable thresholds of MBF must be met to achieve these goals. Thresholds have been identified and combined into a novel metric known as coronary flow capacity (CFC). Outcome data have demonstrated significant reduction in death and MI when CFC is used to select for revascularization versus medical therapy [41, 42]. We have demonstrated that artery-specific reduced CFC also predicts improved perfusion metrics after revascularization that is not realized with angiographically guided revascularization [43]. As such, researchers and clinicians will require standardization and accuracy across software platforms such these end goals can be achieved. However, in this study, we have demonstrated that not only do SWP differ in performance, but several SWP yield data that are not reliably accurate enough for clinical decision making.

#### Limitations

Our study has some limitations. We did not attempt to determine the cause of SWPs’ inaccuracies, as this was outside of the scope of this manuscript. We hope that vendors gain insight from our findings and implement the necessary improvements. Secondly, all studies were processed by nuclear cardiologists who had been trained by the SWPs’ vendors. The studies were not sent to the vendors for processing or troubleshooting. Thus, it is possible that expert vendor staff could have improved implementation of their models on a case-by-case basis. However, as such service is not typically available, our methodology reflects real-world clinical practice. Each patient underwent 3 resting scans, and COV was  ~ 10% for all SWP. Thus, upward bias with associated narrow intra-software variability goes against user error and more likely correctly functioning (albeit erroneous) software. In this manuscript, we focused on “low flow” accuracy because it serves as an excellent reference with narrow margins. We did not assess “high flow” accuracy or “normal” volunteers in this study.

## Conclusions

There is significant software-based variability in the assessment of resting myocardial blood flow of transmural scar. Contemporary 3D-PET with ^82^Rb using the HeartSee software package accurately (median 0.26 mL/min/g, consistent with established values derived invasively and non-invasively) and precisely (COV = 0.07) quantified rMBF in regions of TMS. Emory-O yielded somewhat similar results, though in ~ 5% of the resting scans, rMBF was higher than expected for TMS. The other SWPs tested, including 4DM, 4DM-FDV, Cedars, and Emory-V, consistently yielded values higher than expected for transmural scar in 67%, 29%, 59%, and 55% of cases, respectively.

### Supplementary Information

**Additional file 1: Table S1** *p*-values for comparison of Seg-Scar between each software package. Accompanies Fig. 6. **Table S2** *p*-values for comparison of minimum rMBF segments between each SWP. Accompanies Fig. 7. **Table S3** ^82^Rb bolus @50 mL/min versus ^82^Rb slow infusion @20 mL/min of resting MBF in regions/segments of transmural scar by software package. **Table S4** Dates of subject PET scans, sizes of infarct and rMBF of ROI of infarct.

## Data Availability

The datasets used and/or analyzed during the current study are available from the corresponding author on reasonable request.

## Abbreviations

PET: Positron emission tomography
rMBF: Resting myocardial blood flow
ROI: Region of interest
SWP: Software package
TMS: Transmural Scar
