# Supplementary material for: Assessment of resting myocardial blood flow in regions of known transmural scar to confirm accuracy and precision of 3D cardiac positron emission tomography
Source: EJNMMI Res. 2023 Sep 27;13:87. doi: 10.1186/s13550-023-01037-7 (PMC10522549; doi:10.1186/s13550-023-01037-7)
Supplement: Supplementary file 1 — Additional file 1: Table S1 p-values for comparison of Seg-Scar between each software package. Accompanies Fig. 6. Table S2 p-values for comparison of minimum rMBF segments between each SWP. Accompanies Fig. 7. Table S3 82Rb bolus @50 mL/min versus 82Rb slow infusion @20 mL/min of resting MBF in regions/segments of transmural scar by software package. Table S4 Dates of subject PET scans, sizes of infarct and rMBF of ROI of infarct. [file 13550_2023_1037_MOESM1_ESM.docx]

Supplemental Tables

Supplemental Table 1 - p-values for comparison of Seg-Scar between each software package.

|  | HS-ROI | HS seg | 4DM | 4DM-FDV | Cedars | Emory-V | Emory-O |
| --- | --- | --- | --- | --- | --- | --- | --- |
| HS-ROI | NA | 0.004 | <0.001 | <0.001 | <0.001 | <0.001 | <0.001 |
| HS-Seg | 0.004 | NA | <0.001 | <0.001 | <0.001 | <0.001 | 0.006 |
| 4DM | <0.001 | <0.001 | NA | <0.001 | **0.135** | <0.001 | <0.001 |
| 4DM-FDV | <0.001 | <0.001 | <0.001 | NA | <0.001 | 0.003 | 0.004 |
| Cedars | <0.001 | <0.001 | **0.135** | <0.001 | NA | <0.001 | <0.001 |
| Emory-V | <0.001 | <0.001 | <0.001 | 0.003 | <0.001 | NA | <0.001 |
| Emory-O | <0.001 | 0.006 | <0.001 | 0.004 | <0.001 | <0.001 | NA |

Accompanies Figure 6

Supplemental Table 2 - p-values for comparison of minimum rMBF segments between each SWP.

|  | HS | HS seg | 4DM | 4DM-FDV | Cedars | Emory-V | Emory-O |
| --- | --- | --- | --- | --- | --- | --- | --- |
| HS-ROI | NA | <0.001 | <0.001 | <0.001 | <0.001 | <0.001 | <0.001 |
| HS-Seg | <0.001 | NA | <0.001 | 0.013 | <0.001 | <0.001 | **0.273** |
| 4DM | <0.001 | <0.001 | NA | <0.001 | 0.026 | <0.001 | <0.001 |
| 4DM-FDV | <0.001 | <0.001 | <0.001 | NA | <0.001 | <0.001 | **0.082** |
| Cedars | <0.001 | <0.001 | 0.026 | <0.001 | NA | 0.104 | <0.001 |
| Emory-V | <0.001 | <0.001 | <0.001 | <0.001 | 0.104 | NA | <0.001 |
| Emory-O | <0.001 | **0.273** | <0.001 | **0.082** | <0.001 | <0.001 | NA |

Accompanies Figure 7

| Software Package | ^82^Rb Bolus @50mL/min  (n=29) | ^82^Rb Slow Infusion @20mL/min  (n=31) | p-value |
| --- | --- | --- | --- |
| HeartSee ROI | 0.25  [0.22-0.30] | 0.28  [0.24-0.32] | 0.161 |
| HeartSee Seg | 0.27  [0.25-0.38] | 0.30  [0.26-0.40] | 0.257 |
| 4DM | 0.79  [0.50-0.98] | 0.68  [0.53-1.02] | 0.826 |
| 4DM-FDV | 0.43  [0.34-0.54] | 0.41  [0.34-0.53] | 0.907 |
| Cedars | 0.63  [0.50-0.85] | 0.71  [0.51-0.84] | 0.736 |
| Emory-V | 0.53  [0.43-0.62] | 0.49  [0.43-0.60 | 0.394 |
| Emory-O | 0.37  [0.30-0.41] | 0.36  [0.30-0.43] | 0.895 |

Supplemental Table 3 – ^82^Rb bolus @50 mL/min vs. ^82^Rb slow infusion @20 mL/min of resting MBF in regions/segments of transmural scar by software package

Supplemental Table 4

| Patient | Duration of Scar  (years) | Date of first prior PET | Date of Study PET | Date difference between PET scans  (years) | Size of TMS on prior PET  (% of LV) | Size of TMS on Study PET (% of LV) | ROI-Scar rMBF  (mL/min/g) prior PET | ROI-Scar rMBF  (mL/min/g) Study PET |
| --- | --- | --- | --- | --- | --- | --- | --- | --- |
| 1 | 8 | 1/17/2013 | 1/20/2022 | 9.01 | 22 | 23 | 0.33 | 0.29 |
| 2 | 8 | 3/27/2020 | 1/21/2022 | 1.82 | 24 | 30 | 0.23 | 0.20 |
| 3 | 4 | 9/23/2019 | 1/25/2022 | 2.34 | 14 | 19 | .4 | 0.31 |
| 4 | 4 | 1/03/2019 | 1/25/2022 | 3.06 | 19 | 18 | .25 | 0.34 |
| 5 | 3 | 4-5-2019 | 1/28/2022 | 2.82 | 26 | 28 | 0.36 | 0.33 |
| 6 | 19 | 1-25-2019 | 1/28/2022 | 3.01 | 24 | 27 | 0.25 | 0.24 |
| 7 | 9 | 8-20-13 | 1/31/2022 | 8.45 | 51 | 53 | 0.31 | 0.21 |
| 8 | 10 | 7-30-2015 | 1/31/2022 | 6.51 | 16 | 15 | .34 | 0.34 |
| 9 | 16 | 8-27-2019 | 2/2/2022 | 2.44 | 21 | 20 | 0.31 | 0.27 |
| 10 | 20 | 9-27-2016 | 2/7/2022 | 5.37 | 19 | 21 | 0.35 | 0.31 |
| 11 | 6 | 5-16-2016 | 2/9/2022 | 5.74 | 31 | 28 | .35 | 0.34 |
| 12 | 5 | 12-27-2017 | 2/10/2022 | 4.13 | 30 | 30 | .29 | 0.23 |
| 13 | 7 | 3-9-2015 | 2/11/2022 | 6.93 | 34 | 34 | .27 | 0.25 |
| 14 | 2 | 1-26-2021 | 2/25/2022 | 1.08 | 18 | 20 | 0.30 | 0.22 |
| 15 | 18 | 1-25-2021 | 5/11/2022 | 1.29 | 25 | 23 | 0.27 | 0.23 |
| 16 | 6 | 6-30-2016 | 5/24/2022 | 5.9 | 32 | 31 | 0.28 | 0.32 |
| 17 | 1 | 6-16-2021 | 6/22/2022 | 1.02 | 33 | 34 | 0.26 | 0.31 |
| 18 | 3 | 11-4-2019 | 7/28/2022 | 2.73 | 30 | 29 | .23 | 0.20 |
| 19 | 8 | 6-20-2016 | 8/11/2022 | 6.15 | 28 | 31 | .32 | 0.26 |
| 20 | 15 | 4-18-2022 | 8/19/2022 | 0.34 | 19 | 17 | 0.2 | 0.24 |
| Mean | 8.6+/-5.9 | NA | NA | 4.0+/-2.6 | 26+/-8^*^ | 27+/-9^*^ | 0.30+/-0.05^**^ | 0.27+/-.05^**^ |

* p = 0.842

**p = 0.159
